# The Roles of Impurities and Surface Area on Thermal Stability and Oxidation Resistance of BN Nanoplatelets

**DOI:** 10.3390/nano14070601

**Published:** 2024-03-28

**Authors:** Nikolaos Kostoglou, Sebastian Stock, Angelos Solomi, Damian M. Holzapfel, Steven Hinder, Mark Baker, Georgios Constantinides, Vladislav Ryzhkov, Jelena Maletaskic, Branko Matovic, Jochen M. Schneider, Claus Rebholz, Christian Mitterer

**Affiliations:** 1 Department of Materials Science, Montanuniversität Leoben, 8700 Leoben, Austria; a.solomi@hotmail.com (A.S.); claus@ucy.ac.cy (C.R.); christian.mitterer@unileoben.ac.at (C.M.); 2 Department Physics, Mechanics and Electrical Engineering, Montanuniversität Leoben, 8700 Leoben, Austria; sebastian.stock@unileoben.ac.at; 3 Department of Mechanical and Manufacturing Engineering, University of Cyprus, Nicosia 2109, Cyprus; 4 Materials Chemistry, RWTH Aachen University, 52074 Aachen, Germany; holzapfel@mch.rwth-aachen.de (D.M.H.); schneider@mch.rwth-aachen.de (J.M.S.); 5 Department of Mechanical Engineering Sciences, University of Surrey, Guildford GU2 7XH, UK; s.hinder@surrey.ac.uk (S.H.); m.baker@surrey.ac.uk (M.B.); 6 Department of Mechanical Engineering and Materials Science and Engineering, Cyprus University of Technology, Lemesos 3036, Cyprus; g.constantinides@cut.ac.cy; 7 Research School of High-Energy Physics, Tomsk Polytechnic University, 634050 Tomsk, Russia; ryzhkov@tpu.ru; 8 Vinča Institute of Nuclear Sciences—National Institute of the Republic of Serbia, University of Belgrade, 11000 Belgrade, Serbia; jelena.pantic@vinca.rs (J.M.); mato@vin.bg.ac.rs (B.M.)

**Keywords:** nanomaterials, nanostructures, hexagonal boron nitride, nanoplatelets, purity, surface area, thermal stability, oxidation resistance

## Abstract

This study considers the influence of purity and surface area on the thermal and oxidation properties of hexagonal boron nitride (h-BN) nanoplatelets, which represent crucial factors in high-temperature oxidizing environments. Three h-BN nanoplatelet-based materials, synthesized with different purity levels and surface areas (~3, ~56, and ~140 m^2^/g), were compared, including a commercial BN reference. All materials were systematically analyzed by various characterization techniques, including gas pycnometry, scanning electron microscopy, X-ray diffraction, Fourier-transform infrared radiation, X-ray photoelectron spectroscopy, gas sorption analysis, and thermal gravimetric analysis coupled with differential scanning calorimetry. Results indicated that the thermal stability and oxidation resistance of the synthesized materials were improved by up to ~13.5% (or by 120 °C) with an increase in purity. Furthermore, the reference material with its high purity and low surface area (~4 m^2^/g) showed superior performance, which was attributed to the minimized reactive sites for oxygen diffusion due to lower surface area availability and fewer possible defects, highlighting the critical roles of both sample purity and accessible surface area in h-BN thermo-oxidative stability. These findings highlight the importance of focusing on purity and surface area control in developing BN-based nanomaterials, offering a path to enhance their performance in extreme thermal and oxidative conditions.

## 1. Introduction

Hexagonal boron nitride (h-BN) nano-sized platelets, commonly known as nanoplatelets or nanoplates, have emerged as a material of significant interest in the field of nanotechnology, owing to their unique thermal, electrical, optical, and mechanical properties and their wide range of applications, including the emerging fields of nanoelectronics, optoelectronics, nanophotonics, thermoelectrics, biosensing, and even therapeutics [1,2,3,4]. h-BN nanoplatelets are characterized by a thin, plate-like morphology with a thickness in the nanometer range, while their length and width can be significantly larger, often in the micron range. This two-dimensional material, which is isostructural to graphene yet composed of different elements, has been the center of attention for its intrinsic properties, including its remarkable thermal conductivity, superior electrical insulation, enhanced mechanical strength, and high chemical stability [1,2]. h-BN nanoplatelets are also known for their exceptional thermal stability and resistance to oxidation [5,6], making them ideal candidates for applications in high-temperature oxidizing environments. In this respect, h-BN nanoplatelets have the potential to be used as effective and high-performance functional components, such as protective coatings, solid lubricants, refractory ceramics, thermal barriers, thermal interface materials, radiation shields, dielectric layers, and many more, in automotive, aerospace, semiconductor, and other industries.

While the bulk properties of h-BN nanoplatelets have been studied in the literature [7,8,9,10], there is a notable gap in understanding how intrinsic factors, particularly sample purity and accessible surface area, can influence their thermal stability and oxidation resistance properties. Both purity and surface area seem to be critical parameters in determining the performance and applicability of BN materials in general, as hinted in previous research studies [6,11], yet their precise roles remain under-explored. Notably, Wang and colleagues [11] studied BN nanosheets, nanoplates, and nanocrystals of various crystallinities and surface areas, observing higher thermal stability and oxidation resistance for the BN nanostructures with higher crystallinity and lower surface area. Kostoglou and co-workers reported a higher oxidation resistance temperature for non-porous, low-surface area, and high-purity h-BN nanoplatelets [5] compared with nanoporous, high-surface area, and high-purity h-BN nanoplatelets [6]. Zhu et al. [12] synthesized nanoporous and high-surface area h-BN flakes, which exhibited comparable oxidation resistance to the high-surface area BN materials presented by Wang et al. [11] and Kostoglou et al. [6]. Therefore, combining the observations of these studies suggests that BN materials with lower porosity and surface area tend to exhibit enhanced thermal stability and oxidation resistance.

This study endeavors to bridge the gap in comprehending the impact of purity and surface area on the thermo-oxidative behavior of BN materials by systematically investigating three h-BN powders composed of nanoplatelets, produced with different levels of purity and variations in surface area (by two orders of magnitude), through a combination of advanced characterization techniques and high-resolution thermal analysis. The h-BN nanoplatelets were synthesized by a two-step thermal treatment of boric acid and urea mixtures under inert and oxidizing gas atmospheres and were thoroughly characterized for their morphology, microstructure, purity, surface chemistry, elemental composition, skeletal density, pore structure, and surface area by scanning electron microscopy (SEM), X-ray diffraction (XRD), Fourier-transform infrared radiation (FT-IR), X-ray photoelectron spectroscopy (XPS), helium (He) pycnometry and nitrogen (N_2_) adsorption/desorption measurements at 77 K. Their thermal stability and oxidation behavior were studied by combining thermal gravimetric analysis (TGA) and differential scanning calorimetry (DSC) under a controlled oxidizing environment. A commercially available h-BN material of high purity and low surface area (~4 m^2^/g) was also characterized and employed for reference purposes. Last but not least, this research aims not only to deepen our understanding of BN nanoplatelet behavior in high-temperature oxidizing environments but also to elucidate the underlying mechanisms driving their performance. The following sections detail the synthesis methods and analytical techniques employed in this study, followed by a discussion of the characterization results and their implications in the broader context of BN nanomaterial research.

## 2. Materials and Methods

### 2.1. Commercial BN Reference

A BN powder of 99.5% purity and an average particle size of 10 μm, according to its technical specifications, was purchased from GoodFellow Cambridge Ltd. (product code B516011/9; Huntington, UK). This sample was used as a reference high-purity BN material in this study and is hereafter denoted as BN-GF.

### 2.2. Synthesis Method

Three different BN powders were prepared by adapting the synthesis method introduced by Matovic and co-workers [6,13], specifically by employing three distinct annealing temperatures. In a typical synthesis procedure, three mixtures of boric acid (H_3_BO_3_; Zorka Šabac Co., Šabac, Serbia) and urea (CO(NH_2_)_2_; Fisher Scientific, Waltham, MA, USA) with molar ratios of 1:5 were dissolved in ethanol (C_2_H_6_O) and stirred for 3 h using a magnetic stirrer. This particular molar ratio was selected to optimize the BN synthesis and enhance the reaction yield through the presence of a nitrogen-rich environment, as supported by stoichiometric considerations and empirical evidence. After drying in air at 80 °C, the three obtained powders were annealed at 750, 800, and 850 °C, respectively, under an inert N_2_ atmosphere for 16 h using a heating rate of 4 °C/min. After cooling down to room temperature, all three powders were then calcined in air at 600 °C for 2 h using a heating rate of 10 °C/min with the aim to burn off any remaining organic matter and to oxidize and eliminate any B-containing compounds, except BN, still present following pyrolysis. Next, the powders were centrifuged five times (i.e., four times in H_2_O and once in C_2_H_6_O) for 5 min at 4200 rpm to remove any residual impurities. The residues were then dried again in air at 80 °C, and the three versions of BN powders were obtained, denoted hereafter as BN-V1 (annealed at 750 °C), BN-V2 (annealed at 800 °C) and BN-V3 (annealed at 850 °C), respectively.

### 2.3. Characterization Methods

Surface morphology was studied by collecting high-resolution images with an SEM device (Quanta 200; FEI, Hillsboro, OR, USA) using an acceleration voltage in the 10–20 kV range and a working distance of 10 mm. Prior to the SEM investigations, all powder samples were sputter coated with Ag in an inert Ar atmosphere (SC7640; Quorum Technologies, East Sussex, UK) to improve surface conductivity and avoid potential charging effects during imaging. SEM images were collected at magnifications in the range of ×100 to ×20,000.

Microstructure and purity were investigated by XRD measurements using an X-ray diffractometer (D8 Advance; Bruker-AXS, Karlsruhe, Germany) equipped with Cu Kα radiation (~0.154 nm wavelength), operating at a 40 kV voltage and a 40 mA current. XRD patterns were recorded using the Bragg–Brentano configuration with a continuous scan speed between the diffraction angles 2θ of 10–90°, advancing at a 0.01° step width and a 0.5°/min scanning rate. For each sample, the interlayer distance (d) was calculated from the (002) reflection using Bragg’s law [14], while the crystallite thickness (L_c_) and lateral size (L_a_) were estimated from the (002) and (100) reflections, respectively, using Scherrer equation [15]. The graphitization index (GI) was estimated by the integrated areas (A) of the (100), (101), and (102) reflections using the ratio (A_(100)_ + A_(101)_)/A_(102)_, based on the definition given by Thomas et al. [16].

Surface chemistry and elemental composition analysis were performed using FT-IR and XPS. FT-IR spectra were recorded by an FT-IR spectrometer (JASCO-4100; Shanghai, China) in the mid-infrared wavenumber range of 4000–400 cm^−1^. Samples of ~1 mg were mixed with potassium bromide, and the prepared mixture was subjected to high-pressure compaction to form thin translucent discs. XPS measurements were conducted with an X-ray photoelectron spectrometer system (K-Alpha+; ThermoFisher Scientific, East Grinstead, UK), which uses a monochromated Al Kα X-ray source (1486.6 eV photon energy) and a ~400 µm radius X-ray spot. Wide-scan survey spectra and high-resolution core-level spectra for the different components were collected with a pass energy of 200 eV and 50 eV, respectively. To compensate for potential charging effects that might occur during the measurement process, all acquired spectra were charge-referenced to the C1s peak at 285.0 eV [17]. Quantification of the chemical compositions was performed using the peak areas of the high-resolution core-level spectra and applying instrument-modified Wagner sensitivity factors, including a non-linear Shirley-type background subtraction.

The loose bulk densities of the studied powders were estimated by following the respective standard of the American Society for Testing and Materials (ASTM D7481-18 [18]). The powders were gently poured into a container, and their mass was measured using a gravimetric balance after completely filling the container. The masses of the powders were then divided by the volume of the container to calculate the loose bulk densities. The skeletal densities were determined by helium (He) pycnometry using a pycnometer device (Ultrapyc 5000; Anton-Paar QuantaTec, Boynton Beach, FL, USA). The experiments were performed at 20 °C with He pressurization cycles between 0.034 bar and 1.24 bar. Prior to the measurement, the sample holder was calibrated using a reference silicon sphere of known volume. It should be noted that for every sample, the average value was determined from five separate runs, which exhibited a variance of less than 0.1%, ensuring high precision in these measurements.

Nanopore structure and surface area analyses were carried out by N_2_ adsorption and desorption measurements at 77 K using a manometric gas sorption analyzer (Autosorb iQ^3^; Anton-Paar QuantaTec, Boynton Beach, FL, USA). He and N_2_ gases of ultra-high purity (99.999%) were employed for void volume calculations and gas sorption analysis, respectively, while a liquid N_2_ bath was used for cooling purposes. Prior to the tests, samples of ~350 mg were thoroughly degassed under vacuum (10^−6^ mbar) at 250 °C for 24 h to make the surface more accessible by removing physisorbed species. The specific surface area (SSA) was calculated using the multi-point Brunauer–Emmet–Teller (BET) method, following the BET consistency criteria of the International Standard Organization (ISO 9277:2022 [19]). The micropore SSA and volume were calculated by applying the statistical thickness (t-plot) method [20] to adsorption data in the relative pressure (P/P_0_) range of 0.2–0.5.

Thermal stability and oxidation resistance were explored by combining TGA and DSC analyses using a thermo-microbalance (STA 449 C Jupiter^®^; Netzsch, Selb, Germany) using a continuous heating mode up to 1350 °C, a 10 °C/min heating rate, and a 15 sscm Ar-O_2_ (80–20) gas mixture flow. Samples of ~10 mg were placed in alumina crucibles, while buoyancy effects were considered by carrying out a blank measurement. Initially, the powders were degassed under vacuum (10^−4^ mbar) for 2 h at 200 °C to remove any physisorbed gases or moisture from their surface. Prior to TGA/DSC analysis, purging was applied to remove any residual contaminants from inside the furnace. For temperature and sensitivity calibration of the device, high purity (≥99.9%) standards (Zn, Sn, Al, Ag, Au, and Pd) were melted.

## 3. Results and Discussion

### 3.1. Surface Morphology

**Figure 1:**
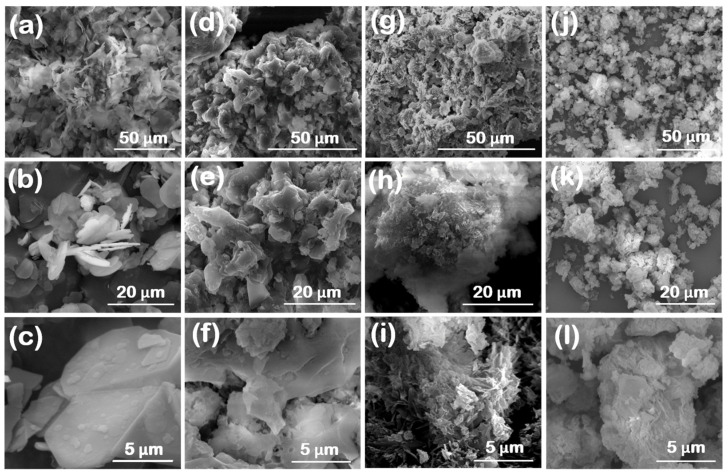
SEM images at different magnifications for (**a**–**c**) reference BN-GF and synthesized (**d**–**f**) BN-V1, (**g**–**i**) BN-V2 and (**j**–**l**) BN-V3 materials.

SEM images at different magnifications for the reference and synthesized BN powders are displayed in Figure 1. The reference BN-GF sample (Figure 1a–c) demonstrates a platelet-based particle morphology characteristic of this type of material [5,9,10,21]. An assemblage of platelets can be clearly seen at lower magnifications (Figure 1b). In contrast, the individual platelets seem to span up to several micrometers in width, as observed at higher magnifications (Figure 1c). For the synthesized materials, the surface morphology becomes more and more defective by moving from the BN-V1 (Figure 1d–f) to the BN-V2 (Figure 1g–i) and finally to the BN-V3 (Figure 1j–l) sample. The relatively smooth platelet morphology of the BN-V1 sample, observed at higher magnifications (Figure 1f), is replaced by a rougher surface in the cases of BN-V2 (Figure 1i) and BN-V3 (Figure 1l). As can be seen from Figure 1l, the BN-V3 sample consists of micrometer-sized agglomerates, which, however, seem to be composed of much smaller platelets and crystals. The presence of these finer particles hints towards higher external surface areas available for the BN-V2 and BN-V3 powders.

### 3.2. Microstructure and Purity

**Figure 2:**
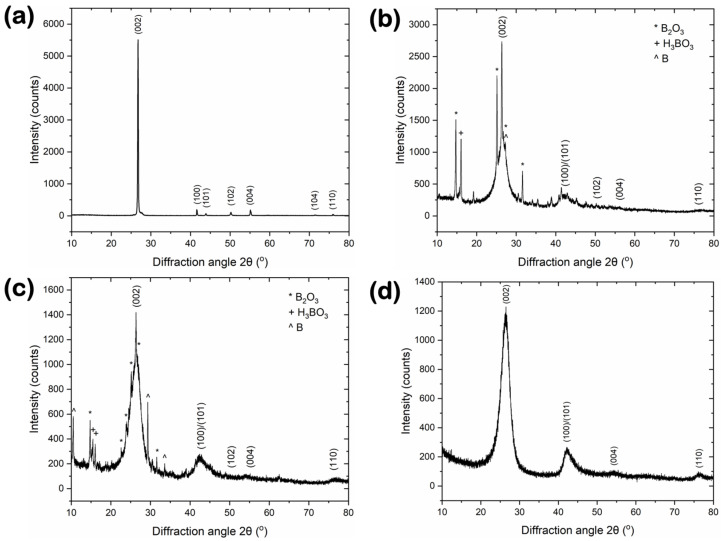
X-ray diffractograms for (**a**) reference BN-GF and synthesized (**b**) BN-V1, (**c**) BN-V2, and (**d**) BN-V3 materials.

XRD data for the reference and synthesized BN materials are shown in Figure 2. The X-ray diffractograms reveal similar features for the three synthesized BN materials (Figure 2b–d) related to the hexagonal BN crystalline structure, according to the International Centre for Diffraction Data (ICDD) card no. 34-0421, including the dominant (002) reflection at 2θ ~ 26.4° (for all samples), followed by much less intense peaks at ~41.4° (BN-V1), ~41.8° (BN-V2) and ~42.1° (BN-V3), corresponding to the (100)/(101) reflections, respectively. However, these peaks are broader (i.e., larger full widths at half maximum) and show a lower signal-to-noise ratio than the ones of the reference BN-GF sample (Figure 2a), highlighting the fact that the synthesized samples are less crystalline, could have more defects, and probably consist of smaller crystallites. In addition, for the bulk BN counterpart, these peaks are located at slightly higher 2θ angles (i.e., ~26.7° for (002), ~41.6° for (100), and ~43.9° for (101)), while smaller peaks can be seen clearly at ~50.2° and ~55.2°, corresponding to the (102) and (004) reflections, respectively.

A detailed quantitative analysis of the XRD patterns allowed the calculation of important structural properties such as interlayer distance, crystallite thickness, crystallite lateral size, number of layers, and graphitization index (see Table 1). The interlayer spacing for the synthesized samples was estimated at ~0.339 nm in all cases using Bragg’s law, which is 1.5% larger than that of the BN-GF reference (i.e., ~0.334 nm) and 1.8–2.7% larger than the theoretical values for h-BN found in the literature (i.e., 0.330–0.333 nm) [22]. The increased interlayer distance observed for the synthesized BN samples suggests a turbostratic structure, which is associated with smaller crystallite sizes in both the thickness and lateral dimensions. Indeed, the average crystallite thicknesses (L_c_) and lateral sizes (L_a_) for the synthesized BN-V1, BN-V2, and BN-V3 samples were estimated at around 13, 5.6, and 3.2 nm and 50, 38, and 13.5 nm, respectively, using Scherrer’s formula, far smaller than the respective values of the BN-GF reference (i.e., 41.3 nm for L_c_ and 210 nm for L_a_, respectively). Thus, a comparative analysis between the synthesized samples reveals that the L_c_ and L_a_ values of BN-V2 were reduced by ~57% and ~24%, respectively, while the equivalent values of BN-V3 showed more pronounced decreases of ~75% and ~73%, respectively, compared with BN-V1. The average number of BN layers was also estimated at ~39, ~18, and ~9 for BN-V1, BN-V2, and BN-V3, respectively, upon dividing the crystallite thickness to the interlayer distance and adding one layer, while the equivalent number for the BN-GF reference was ~125. According to the estimated graphitization indexes of ~16, ~60, and ~69 for BN-V1, BN-V2, and BN-V3, it can be concluded that the synthesized materials are not as well graphitized and crystalline as the BN-GF reference (i.e., GI of ~1.64). Balint and Petrescu [23] suggested that BN powders with a GI value of ~1.6 exhibit a perfectly graphitic-like crystalline structure, while a maximum GI value of ~50 is considered the upper limit before transitioning to a disordered turbostratic structure, i.e., hexagonally structured BN layers with random orientation to each other [24]. Based on this definition, the structure of the BN-V2 and BN-V3 samples can be classified as turbostratic.

Finally, concerning the identification and evaluation of impurity phases, the XRD patterns of the BN-V1 and BN-V2 samples revealed crystalline residues of H_3_BO_3_, boron trioxide (B_2_O_3_), and elemental B related to the precursors and the synthesis procedure (see Section 2.2), thus indicating partially incomplete BN synthesis in these two cases. Instead, such admixtures were not detectable in the case of the synthesized BN-V3 and BN-GF reference samples, thus indicating their superior BN purity. The lack of impurities in the BN-V3 sample could be related to its higher annealing temperature during synthesis (i.e., 850 °C). It should be noted that similar XRD patterns for BN nanoplatelets were previously reported in the literature [5,6].

In conclusion, the XRD analysis of the synthesized BN materials revealed distinct structural discrepancies in comparison to the reference BN-GF materials, particularly in terms of crystallinity, crystallite size, and impurity content. These structural attributes are critical in determining the behavior of BN materials in high-temperature oxidizing environments, directly affecting their thermal stability and oxidation resistance. Notably, the reductions in crystallinity and crystallite dimensions observed for the synthesized samples are likely to diminish their thermo-oxidative performance. This is because decreased crystallinity is associated with increased defect sites within the crystal structure and the fact that smaller crystallites exhibit higher surface-to-volume ratios, thereby increasing the reactivity of these materials. Additionally, the detected impurities in the BN-V1 and BN-V2 samples could further undermine their oxidation resistance, making them more susceptible to degradation in oxidative conditions.

### 3.3. Surface Chemistry and Chemical Composition

**Figure 3:**
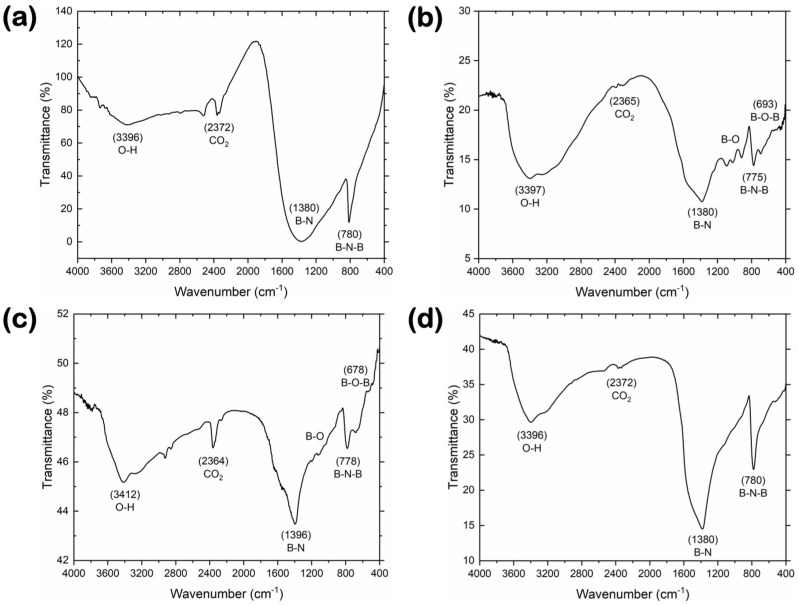
FT-IR spectra for (**a**) reference BN-GF and synthesized (**b**) BN-V1, (**c**) BN-V2, and (**d**) BN-V3 materials.

FT-IR and XPS measurements were performed to elucidate the surface chemistry characteristics of the BN materials. Smoothed FT-IR spectra for the reference and synthesized BN materials in the mid-infrared region are depicted in Figure 3. The three synthesized samples (Figure 3b–d) showed comparable FT-IR spectra, with the signal-to-noise ratio increasing upon moving from the BN-V1 to the BN-V3 sample. The characteristic phonon vibration modes associated with the sp^2^-hybridized bonds of hexagonal BN are observed at the wavenumber regions of ~775–780 and ~1380–1396 cm^−1^. More specifically, the sharper and less intense band corresponds to the B–N–B out-of-plane bending vibration, while the wider and stronger band is related to the in-plane B–N stretching vibration [25,26]. Furthermore, for the cases of BN-V1 and BN-V2 samples, a small “shoulder” appears at ~678–693 cm^−1^, next to the B–N–B band, which indicates the B–O–B bending vibration of B_2_O_3_ and/or H_3_BO_3_, along with a series of features located at ~917, ~1021 and ~1091 cm^−1^, which could be related to the B–O stretching vibration of B_2_O_3_ and/or H_3_BO_3_ [27,28]. This supports the findings of the XRD analysis, where crystalline residues of H_3_BO_3_ and B_2_O_3_ were detected for the BN-V1 and BN-V2 samples. It should be noted that for all samples, the weakest band observed at ~2364–2372 cm^−1^ can be attributed to the atmospheric CO_2_, while the broad band formed around ~3400 cm^−1^ is usually assigned to the O–H vibration of the adsorbed H_2_O. The high-purity reference BN-GF material (Figure 3a) demonstrated similar features to the lab-synthesized BN-V3 sample, with a more pronounced B–N band and a less pronounced O–H band. This also agrees well with XRD data, which highlighted the higher purity of the BN-GF and BN-V3 samples.

**Figure 4:**
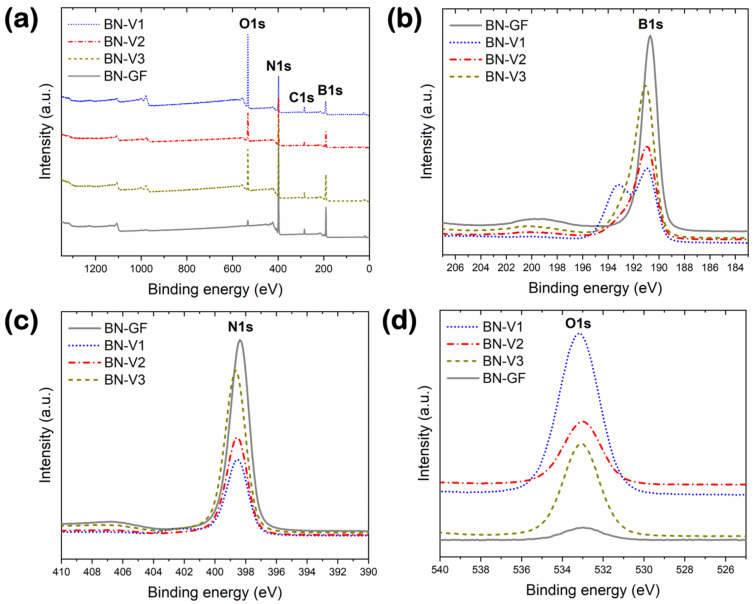
(**a**) XPS survey spectra and high-resolution spectra for (**b**) B 1s, (**c**) N 1s, and (**d**) O 1s peaks of reference (BN-GF) and synthesized (BN-V1, BN-V2, and BN-V3) materials.

XPS data for the reference and synthesized BN materials are presented in Figure 4. The XPS survey spectra (Figure 4a) revealed that all samples contained the elements of B, N, O, and C. Minor traces of Ca and Si were also detected for the reference BN-GF and the synthesized BN-V1 samples. Table 2 summarizes the elemental compositions of the BN materials presented in this study, as determined by XPS analysis. The reference BN-GF sample showed the highest B and N contents among all the samples, together with the lowest O content, followed by BN-V3, BN-V2, and BN-V1, in that order. By moving from BN-V1 to BN-V3, the B and N contents increased (by almost a factor of two for N), while the O content decreased by more than half. Compared with BN-GF, the synthesized samples show higher B/N ratios and lower B/O ratios. This difference stems from the presence of B_2_O_3_ and H_3_BO_3_, further explained in the following paragraph. The sums of the B and N contents correspond to 93.4 at.%, 58.1 at.%, 77.8 at.%, and 79.4 at.% for BN-GF, BN-V1, BN-V2, and BN-V3, respectively, which does not necessarily indicate the BN purity percentages due to the presence of other B-containing compounds. In all synthesized BN samples, the C content ranged between ~4 and ~6 at.%, while a similar C content was observed for the BN reference sample. This residual C could be related to the synthesis precursors (i.e., CO(NH_2_)_2_) and/or adventitious carbon contamination from the atmosphere on the surface of these materials.

High-resolution spectra of B 1s, N 1s, and O 1s for the reference and synthesized BN materials are demonstrated in Figure 4b, Figure 4c, and Figure 4d, respectively. The B 1s peak (Figure 4b) appears at 190.7 eV for the high-purity BN-GF reference, agreeing well with the findings of Baker et al. [29], while for the synthesized samples, the B 1s peak corresponding to BN is found at slightly higher binding energies, being 191.1 ± 0.1 eV for all three samples. This higher binding energy for the BN-V1 to BN-V3 samples is most likely due to the presence of O-containing species being bonded to the BN platelets at defect sites. For the BN-V1 sample, a higher binding energy peak at 193.2 eV is also observed, which could be attributed to boron’s native oxide, B_2_O_3_, and/or boric acid, H_3_BO_3_ [30]. This agrees with the higher O concentration observed for BN-V1 in Table 2 and the stronger B_2_O_3_ and H_3_BO_3_ peaks in the BN-V1 X-ray diffractogram (see Figure 4b). The N 1s peaks (Figure 4c) are observed at 398.4 eV for the BN-GF reference, while the N 1s peaks for the synthesized samples are found at similar binding energies, i.e., 398.5 eV for BN-V1, 398.6 eV for BN-V2 and 398.7 eV for BN-V3, in good agreement with Baker et al. [29]. Finally, the O 1s peak (Figure 4d) is observed to possess similar binding energy values for all BN samples, i.e., 533.0 eV for BN-GF, 533.2 eV for BN-V1 and 533.1 eV for both BN-V2 and BN-V3 samples. These XPS data are consistent with the findings of the XRD and FT-IR studies and further confirm the presence of admixtures in the BN-V1 sample.

### 3.4. Powder Density, Pore Structure, and Surface Area

**Figure 5:**
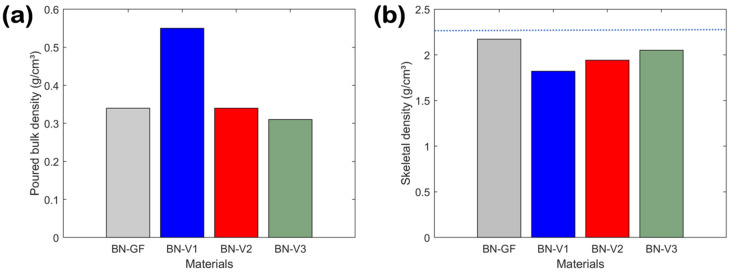
(**a**) Loose bulk densities and (**b**) skeletal densities for reference (BN-GF) and synthesized (BN-V1, BN-V2 and BN-V3) materials. The blue dotted line represents the theoretical density of bulk h-BN (i.e., 2.27 g/cm^3^).

The loose bulk densities and skeletal densities of the reference and synthesized BN powder materials are given in Figure 5. The loose bulk density refers to the mass per unit volume of bulk material, such as powders, granules, or pellets, after it has been poured into a container. It is calculated by simply pouring the material into a known volume and measuring the mass. This includes the volume of the particles and the volume of the void spaces in between them. This type of density is influenced by factors such as particle size and shape, which determine how easily the particles can rearrange to fill the available space. This physical property is important in terms of material handling, transportation, storage, quality control, cost calculation, and safety. The loose bulk densities of all studied BN materials are shown in Figure 5a. These values decrease by almost a factor of two upon moving from the BN-V1 sample (~0.55 g/cm^3^) to the BN-V3 sample (~0.31 g/cm^3^). It should be noted that the BN-V2 sample exhibits the same loose bulk density as the reference BN-GF sample (i.e., ~0.34 g/cm^3^). The transitions from BN-V1 to BN-V2 and from BN-V1 to BN-V3 resulted in a relative decrease in loose bulk density by ~38.2% and ~43.6%, respectively. The observed reduction in loose bulk density with each synthesized BN sample suggests variations in structural characteristics, such as increased porosity or differences in particle packing efficiency.

On the other hand, skeletal density, also known as true density, describes the density of only the material’s solid skeleton, excluding the pores and void spaces within the material. This is calculated by dividing the material’s mass by its skeleton volume. The latter is measured using techniques, such as gas pycnometry, that exclude the internal pore volume. This type of density reflects only the material’s solid phase and is independent of particle size and shape. This physical property is crucial for understanding and predicting other material characteristics, such as chemical composition/purity, porosity, mechanical strength, thermal conductivity, and electrical resistance. The skeletal densities of all studied BN materials, as determined by He pycnometry, are presented in Figure 5b. These values are gradually increasing by moving from BN-V1 (~1.82 g/cm^3^) to BN-V2 (~1.94 g/cm^3^) and then to BN-V3 (~2.05 g/cm^3^). The progressions from BN-V1 to BN-V2 and from BN-V1 to BN-V3 reflected a relative increase in skeletal density by ~6.6% and ~12.6%, respectively. The high-purity BN-GF reference, as suggested from its technical specifications (see Section 2.1), showed the highest value of ~2.17 g/cm^3^, which lies much closer to the theoretical density of bulk h-BN (i.e., 2.27 g/cm^3^) [31].

**Figure 6:**
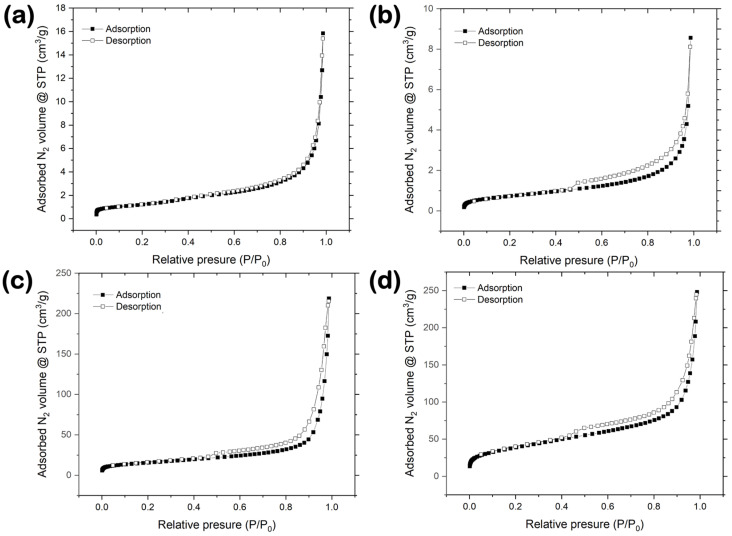
Ν_2_ adsorption and desorption isotherms collected at 77 K for (**a**) reference BN-GF and synthesized (**b**) BN-V1, (**c**) BN-V2, and (**d**) BN-V3 materials.

N_2_ gas adsorption and desorption isotherms were collected at 77 K for reference, and synthesized BN materials can be seen in Figure 6. The reference BN-GF sample (Figure 6a) exhibited a fully reversible N_2_ adsorption/desorption behavior, characteristic of macroporous and/or non-porous materials, Type II according to the classification of the International Union of Pure and Applied Chemistry (IUPAC) [32]. Instead, the synthesized BN-V1 (Figure 6b), BN-V2 (Figure 6c), and BN-V3 (Figure 6d) samples demonstrated comparable but non-reversible N_2_ adsorption/desorption behavior with the characteristic formation of a hysteresis loop at P/P_0_ > 0.45, which is associated with N_2_ capillary condensation in mesopores (i.e., pore widths of 2–50 nm), a phenomenon well-established in the literature and widely known within the gas sorption community [33,34]. Due to this adsorption mechanism, which involves a delay in condensation resulting from the metastability of the pore fluid, the amounts of N_2_ desorbed are observed to be slightly higher than those adsorbed at equivalent P/P_0_ values in that range (i.e., 0.45 < P/P_0_ < 0.99). The isotherms for the synthesized samples can be classified as Type IV, according to the IUPAC classification, indicative of mesoporous materials. The observed hysteresis loop resembles a Type H3, which is associated with non-rigid aggregates of plate-like particles and can also occur if the pore network contains macropores (i.e., pore widths above 50 nm) that are not fully filled with condensate [32]. In all cases, the adsorption curve does not reach a clear saturation point at P/P_0_ ~ 0.99. Instead, it rises unrestrictedly in a vertical direction, a behavior associated with N_2_ condensation in macropores and/or N_2_ adsorption onto external surfaces [35]. The significantly larger volumes of adsorbed N_2_ at P/P_0_ ~ 1 observed in the BN-V2 and BN-V3 samples (i.e., ~225 and ~250 cm^3^/g at STP, respectively), in comparison to the BN-GF and BN-V1 samples (i.e., ~16 and ~9 cm^3^/g at STP, respectively), indicate that the former materials possess larger external surface areas. Finally, the enhanced N_2_ adsorption behavior at the lower P/P_0_ values (<0.01) for the BN-V3 sample hints towards the presence of small fractions of micropores (i.e., pore widths below 2 nm) [6]. To sum up, it can be concluded that the synthesized BN samples combine mainly features of meso- and macro-porosity.

**Figure 7:**
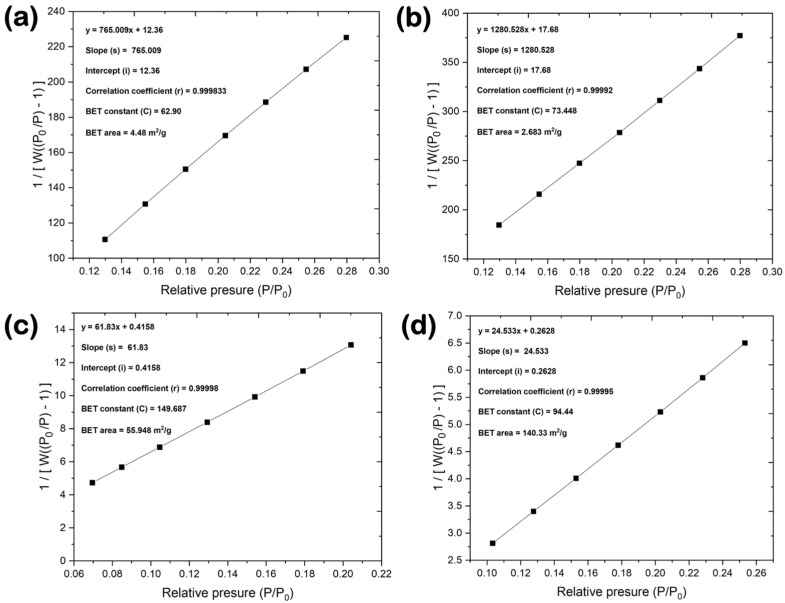
Linear multi-point BET plots within the P/P_0_ range of 0.05–0.3 for (**a**) reference BN-GF and synthesized (**b**) BN-V1, (**c**) BN-V2, and (**d**) BN-V3 materials.

Table 3 highlights the changes in the nanopore structure properties of the different BN powder materials, including BET SSA, micropore SSA, and micropore volume. The low-purity BN-V1 sample showed the lowest BET SSA value (~3 m^2^/g) among all the synthesized samples, which is almost identical to the one of the reference BN-GF sample, as well as no micropore SSA and micropore volume contributions based on the t-plot method. By moving to the BN-V2 sample, the SSA value increased ~19 times, and microporosity was responsible for ~18% of the total SSA, with the remaining percentage being attributed to mesopores, macropores, and external surfaces. Finally, the BN-V3 sample showed the highest BET SSA value (~140 m^2^/g) among all samples (i.e., ~47 times higher than BN-V1 and 2.5 times higher than BN-V2), while microporosity was responsible for ~13% of the total SSA. The herein presented BET SSA values for the BN-V2 and BN-V3 samples are comparable to the ones reported for nanocrystalline h-BN powder prepared by sol-gel polycondensation of resorcinol and formaldehyde in the presence of boric acid (BET SSA ~ 60 m^2^/g) [7] and for few-layer h-BN nanosheets synthesized through a chemical blowing approach (BET SSA ~ 140 m^2^/g) [36] and higher than those recently reported for oxygen-functionalized h-BN nanoplatelets (BET SSA values between ~8 and ~37 m^2^/g) [21]. It should be noted that even though the micropore SSA/BET SSA fraction is slightly higher for the BN-V2 sample, the BN-V3 sample showed a relative increase in the micropore volume by a factor of two. The linear multi-point BET plots for all the BN materials, demonstrating a correlation coefficient of close-to-unity, are presented in Figure 7. The BET constants (C), shown in Figure 7, are related to the energy of adsorption and are calculated from the slope (s) and intercept (i). A higher BET constant typically suggests stronger adsorbate-adsorbent interactions, as observed for the BN-V2 and BN-V3 cases.

### 3.5. Thermal Stability and Oxidation Behavior

The thermal stability and oxidation resistance properties were studied by performing a simultaneous TGA/DSC analysis under a controlled oxidizing atmosphere (80% Ar–20% O_2_). TGA measures the mass of the sample as it is heated, cooled, or held at a constant temperature. As the temperature increases, various components of the sample may volatilize, decompose, or oxidize, leading to a change in mass. DSC measures the amount of energy absorbed or released by the sample as it is heated or cooled. Unlike TGA, which monitors mass changes, DSC provides a heat flow curve that indicates endothermic and exothermic processes.

**Figure 8:**
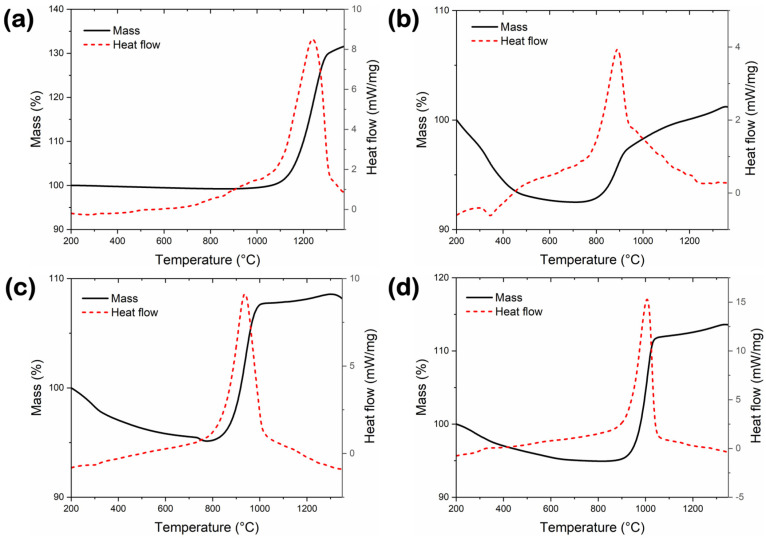
Combined TGA and DSC curves collected under a mixed Ar/O_2_ (80–20) atmosphere for (**a**) reference BN-GF and synthesized (**b**) BN-V1, (**c**) BN-V2, and (**d**) BN-V3 materials.

TGA and DSC heating curves are used as a temperature function for the reference, and synthesized BN materials are combined in Figure 8. All three synthesized BN samples exhibit a comparable trend in their TGA curves (Figure 8b–d), starting with a mass loss, followed by a leveling of the curve, then showing an abrupt mass gain, and finally reaching a saturation point. However, the regions where these processes are observed are shifted to higher temperatures upon increasing the sample purity. In more detail, the sample mass dropped by ~7.5% (up to ~750 °C), ~5% (up to ~800 °C) and ~5% (up to ~900 °C) for BN-V1, BN-V2 and BN-V3, respectively. Since all samples were degassed under vacuum at 200 °C prior to the TGA/DSC analysis, most of the adsorbed surface species (e.g., moisture, trapped gases, etc.) have already been removed. Therefore, this initial mass loss could be related to the dehydration of H_3_BO_3_ that already existed as a residue from the synthesis and/or as a byproduct from the hydration of residual B_2_O_3_, as described in previous relevant studies [6,37,38] and as evidenced by the XRD analysis of the BN-V1 and BN-V2 samples. Upon moving to higher temperatures, the sample mass increased abruptly by ~13% (between ~800–1000 °C) and ~16% (between ~900–1050 °C) for BN-V2 and BN-V3, respectively, and the respective TGA curves reached a plateau. On the contrary, for the lower-purity BN-V1 sample, no clear plateau-like region was formed, and the mass continued to increase up to the completion of the analysis (i.e., by ~9.5% between ~750–1350 °C). This mass gain can be attributed to the decomposition and active oxidation of the BN powders due to oxygen diffusion into the bulk structure and subsequent formation of B_2_O_3_, as reported in other studies [6,35,39]. In this respect, as the BN powders decompose, the B component reacts with the O_2_ of the synthetic Ar-O_2_ atmosphere and produces B_2_O_3_, while molecular N_2_ gas is released based on the chemical reaction 4BN + 3O_2_ → 2B_2_O_3_ + 2N_2_ [37]. It has been suggested that nitric oxide (NO_x_) gases could also be released during this oxidation process [40]. The BN oxidation did not actively proceed by further increasing the temperature, and the excess oxidized mass of the BN-V2 and BN-V3 samples remained stable up to 1350 °C.

The DSC curves of all samples are characterized by a dominant exothermic peak of high intensity, which hints towards a significant thermal event taking place during heating and further supports the BN oxidation process described above. This Gaussian-like peak forms exactly in the range of temperatures where the active oxidation takes place for each sample. The center of this peak shifts to higher temperatures by moving from BN-V1 (i.e., ~885 °C) to BN-V2 (i.e., ~935 °C) and then to BN-V3 (i.e., ~1005 °C). It is worth mentioning that the high-purity and low-SSA BN-GF reference sample (Figure 8a) clearly outperformed all the synthesized materials in terms of thermal stability and oxidation resistance since its TGA curve remains almost fully horizontal up to ~1050 °C. The exothermic DSC peak, in this case, is centered at ~1245 °C, almost 240 degrees higher than the BN-V3 sample that showed the highest purity and highest SSA among the three synthesized samples. To put the TGA/DSC results into context, slightly lower thermal stability (i.e., up to ~1000 °C; DSC peak centered at ~1150 °C) under an oxidizing atmosphere was previously reported by Kostoglou et al. for BN platelets of 200–800 nm width and 30–50 nm thickness with 98% purity and 23 m^2^/g BET SSA [5]. Wang and co-workers [11] synthesized h-BN nanocrystals with 50–250 nm side length, up to 80 nm thickness, and 6–233 m^2^/g SSA values and reported thermal stabilities between 800–1000 °C in the presence of air. Zhu and colleagues [12] synthesized nanoporous h-BN flakes with SSA values of up to ~214 m^2^ /g, which exhibited an oxidation resistance of up to ~800 °C under an oxidizing environment.

To sum up, the thermal stability and oxidation resistance of the herein synthesized BN nanoplatelets improved by ~13.5% (equivalent to a 120 °C increase) upon moving from the lowest purity and lowest SSA (~3 m^2^/g) sample (i.e., BN-V1) to the highest purity and highest SSA (~140 m^2^/g) sample (i.e., BN-V3). Even though higher SSA means more possible surface sites for oxidation, an inverse picture is observed; hence, for these samples in particular, the BN purity seems to play a major role since the presence of admixtures deteriorates their thermal stability and oxidation resistance. However, when comparing the best-performing synthesized sample (i.e., BN-V3) to the BN-GF reference, which is characterized by higher purity, higher crystallinity, and lower SSA, it is evident that the absence of defects and the reduced SSA is crucial for improving the thermal properties of BN materials. This is because fewer reactive sites facilitate oxygen diffusion into the bulk structure and, consequently, oxidation. Therefore, both purity and surface area are important parameters for optimizing and improving the thermo-oxidative stability of BN materials.

## 4. Conclusions

This study presented a comprehensive investigation of the effect of purity and surface area on the thermal and oxidation properties of h-BN nanoplatelets. Through meticulous synthesis via annealing under N_2_ and calcination in air of boric acid–urea mixtures and detailed characterization, including SEM, XRD, FT-IR, XPS, gas pycnometry, gas sorption, and TGA/DSC analyses, the critical roles of sample purity and accessible surface area in determining the performance of BN materials in high-temperature oxidizing environments were elucidated. The results demonstrated a notable increase by ~13.5% (or by 120 °C in absolute values) in the thermal stability and oxidation resistance of h-BN nanoplatelets with higher purity levels (i.e., by progressing from BN-V1 to BN-V3), highlighting the detrimental effects of impurities and underscoring the importance of high purity for optimal performance. Moreover, a comparative analysis between high-purity samples (i.e., reference BNGF vs. synthesized BN-V3), differing in crystallinity (i.e., highly-crystalline BN-GF vs. turbostratic BN-V3) and surface area (i.e., ~4 m^2^/g for BN-GF vs. 140 m^2^/g for BN-V3), revealed that reduced surface area and minimal defects are key factors in improving the thermo-oxidative behavior of BN materials even further (i.e., by 240 °C for BN-GF compared with BN-V3). This can be attributed to the reduced availability of reactive sites for oxygen diffusion and subsequent oxidation in materials with fewer defects and lower surface areas. These findings provide valuable insights for the development and optimization of BN-based materials in general, paving the way to tailor their properties for enhanced performance under extreme thermal and oxidative conditions.

## Figures and Tables

**Table 1:** Structural features for reference (BN-GF) and synthesized (BN-V1, BN-V2, and BN-V3) materials derived by XRD analysis.

Material	2θ_(002)_ [°]	2θ_(100)_ [°]	FWHM_(002)_ [°]	FWHM_(100)_ [°]	d_(002)_ [nm]	L_c(002)_ [nm]	L_a(100)_ [nm]	n [-]	GI [-]
BN-GF	26.74	41.63	0.195	0.083	0.334	41.3	210	125	1.64
BN-V1	26.38	41.41	0.62	0.348	0.339	13	50	39	16
BN-V2	26.34	41.5	1.45	0.457	0.339	5.6	38	18	60
BN-V3	26.38	41.5	2.5	1.29	0.339	3.2	13.5	10	69

**2θ_(002)_:** diffraction angle for the (002) reflection; **2θ_(100)_:** diffraction angle for the (100) reflection; **FWHM_(002)_:** full width at half maximum for the (002) reflection; **FWHM_(100)_:** full width at half maximum for the (100) reflection; **d:** interlayer distance estimated by Bragg’s law in the (002) reflection; **L_c(002)_:** crystallite thickness estimated by Scherrer’s formula in the (002) reflection; **L_a(100)_:** crystallite lateral size estimated by Scherrer’s formula in the (100) reflection; **n:** number of layers estimated by the relationship (L_c_/d) + 1; **GI:** graphitization index estimated by the integrated areas (A) of the (100), (101) and (102) reflections using the ratio (A_(100)_ + A_(101)_)/A_(102)_.

**Table 2:** Elemental compositions of reference (BN-GF) and synthesized (BN-V1, BN-V2, and BN-V3) materials based on XPS data.

Material	Element (at.%)					
	B	N	O	C	Si	Ca
BN-GF	44.3	49.1	2.7	3.8	-	0.1
BN-V1	35.1	23	36.2	5.5	0.2	-
BN-V2	40.2	37.6	18.3	3.9	-	-
BN-V3	40.3	39.1	16.2	4.4	-	-

**Table 3:** Pore structure properties derived from N_2_ adsorption data collected at 77 K for reference (BN-GF) and synthesized (BN-V1, BN-V2, and BN-V3) materials.

Material	S_BET_ [m^2^/g]	S_micro_ [m^2^/g]	S_ext_ [m^2^/g]	V_micro_ [cm³/g]	S_micro_/S_BET_ [%]	S_ext_/S_BET_ [%]
BN-GF	4	0	4	0	0	100
BN-V1	3	0	3	0	0	100
BN-V2	56	10	46	0.005	17.86	82.14
BN-V3	140	18	122	0.010	12.86	87.14

**S_BET_:** Brunauer-Emmett-Teller (BET) specific surface area (SSA); **S_micro_:** micropore SSA derived by the t-plot method; **S_ext_:** sum of mesopore, macropore, and external SSA given as the difference between BET SSA and micropore SSA (S_BET_ − S_micro_); **V_micro_:** micropore volume derived by the t-plot method; **S_micro_/S_BET_:** percentage of micropore SSA to BET SSA; **S_ext_/S_BET_:** percentage of mesopore, macropore, and external SSA to BET SSA.

## Data Availability

The data presented in this study are available on request from the corresponding author.
